# Multisource information fusion method for vegetable disease detection

**DOI:** 10.1186/s12870-024-05346-4

**Published:** 2024-08-02

**Authors:** Jun Liu, Xuewei Wang

**Affiliations:** Shandong Provincial University Laboratory for Protected Horticulture, Weifang University of Science and Technology, Weifang, China

**Keywords:** Vegetables grown in greenhouses, Typical diseases, Detection method, Deep learning, Space-time fusion attention network

## Abstract

Automated detection and identification of vegetable diseases can enhance vegetable quality and increase profits. Images of greenhouse-grown vegetable diseases often feature complex backgrounds, a diverse array of diseases, and subtle symptomatic differences. Previous studies have grappled with accurately pinpointing lesion positions and quantifying infection degrees, resulting in overall low recognition rates. To tackle the challenges posed by insufficient validation datasets and low detection and recognition rates, this study capitalizes on the geographical advantage of Shouguang, renowned as the “Vegetable Town,” to establish a self-built vegetable base for data collection and validation experiments. Concentrating on a broad spectrum of fruit and vegetable crops afflicted with various diseases, we conducted on-site collection of greenhouse disease images, compiled a large-scale dataset, and introduced the Space-Time Fusion Attention Network (STFAN). STFAN integrates multi-source information on vegetable disease occurrences, bolstering the model’s resilience. Additionally, we proposed the Multilayer Encoder-Decoder Feature Fusion Network (MEDFFN) to counteract feature disappearance in deep convolutional blocks, complemented by the Boundary Structure Loss function to guide the model in acquiring more detailed and accurate boundary information. By devising a detection and recognition model that extracts high-resolution feature representations from multiple sources, precise disease detection and identification were achieved. This study offers technical backing for the holistic prevention and control of vegetable diseases, thereby advancing smart agriculture. Results indicate that, on our self-built VDGE dataset, compared to YOLOv7-tiny, YOLOv8n, and YOLOv9, the proposed model (Multisource Information Fusion Method for Vegetable Disease Detection, MIFV) has improved mAP by 3.43%, 3.02%, and 2.15%, respectively, showcasing significant performance advantages. The MIFV model parameters stand at 39.07 M, with a computational complexity of 108.92 GFLOPS, highlighting outstanding real-time performance and detection accuracy compared to mainstream algorithms. This research suggests that the proposed MIFV model can swiftly and accurately detect and identify vegetable diseases in greenhouse environments at a reduced cost.

## Introduction

A recent report revealed that plant diseases account for over one-third of the annual natural losses in agricultural production [1]. Once plants become infected, these diseases can rapidly spread and result in significant production losses. Therefore, early detection and diagnosis of plant diseases are of utmost importance. In the past, agricultural experts were responsible for plant disease detection, requiring extensive professional knowledge. However, this approach proved to be time-consuming, labor-intensive, and prone to errors [2]. The traditional method of manual feature extraction for plant disease detection is complex and inefficient, posing challenges for greenhouse cultivation [3]. Moreover, certain valuable features that are not readily discernible to the naked eye often go unnoticed [4]. Additionally, when confronted with extensive datasets in natural settings, the accuracy of these traditional methods is notably diminished [5]. Fortunately, advancements in artificial intelligence and computer vision technology, particularly in deep learning, offer promising solutions across various fields, including agriculture, surpassing traditional methods. [6, 7].

**Fig. 1:**
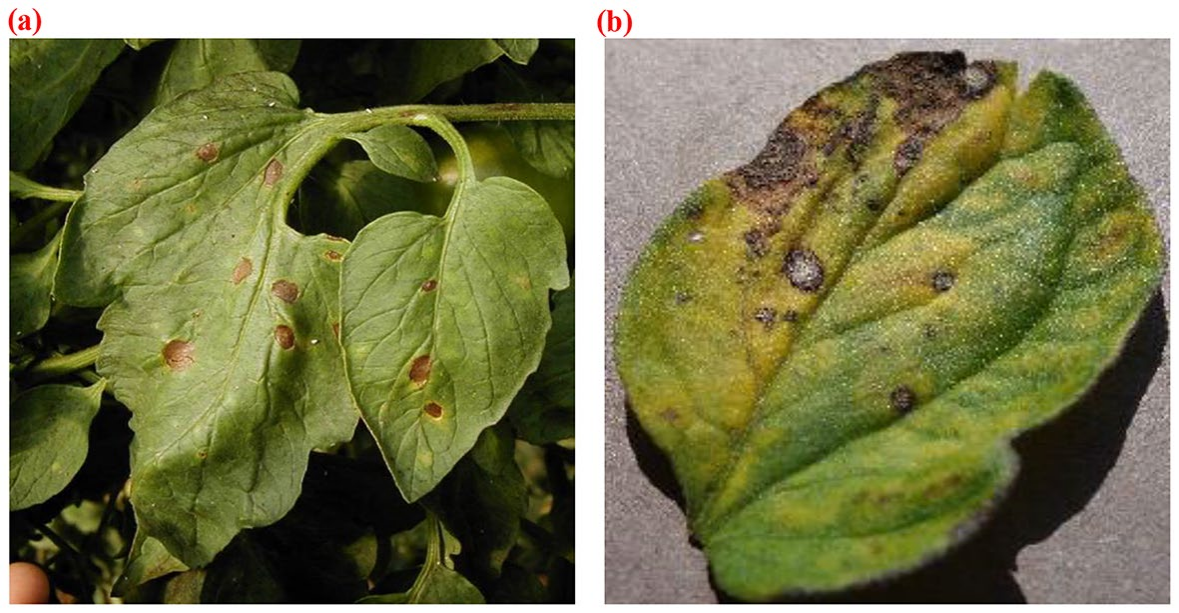
Example images for two environments. (**a**) Images obtained from natural environments (**b**) Images acquired in controlled environments

In recent years, there has been a growing trend towards applying artificial intelligence (AI) methodologies to various aspects of agriculture, including disease detection. Tang et al. [8] incorporated the attention mechanism into ShuffleNet and achieved an impressive accuracy of 99.14% in identifying multiple crop diseases on the PlantVillage dataset. Ni et al. [9] enhanced the ResNet50 network by introducing the concept of dense connections. Their improved model exhibited good performance on the Al Challenger 2018 dataset. Mohapatra et al. [10] introduced custom-CNN for identifying four types of rice leaf diseases. Their model outperformed others with a higher accuracy of 97.47%. Furthermore, researchers have discovered that employing attention mechanisms can enhance the weight of lesion features, thus improving recognition effect. Chen et al. [11] introduced the SE module, significantly improving the model’s sensitivity to channel features. It demonstrated superior performance compared to existing models. He et al. [12] employed a double-layer Faster R-CNN to detect brown planthoppers at different quantities and stages. Wang et al. [13] proposed an S-RPN network, integrating attention mechanisms into residual networks. On their self-built AgriPest21 dataset, they obtained an average accuracy (mAP) of 78.7%. Jiao et al. [14] proposed an adaptive feature fusion module within FPN to extract more comprehensive pest features, along with an adaptive enhancement module to reduce information loss. Li et al. [15] put forward the DAC-YOLOv4 algorithm, which accurately detects strawberry powdery mildew. This method successfully identifies diseased leaves and areas even in complex backgrounds and calculates the disease index based on the incidence of strawberry powdery mildew, providing valuable references for subsequent treatments. Sun et al. [16] proposed VegDenseCap, utilizing vegetable leaf images as input. The disease features are then described in natural language, achieving an average accuracy of 88.0% (mAP). Additionally, Li et al. [17] improved YOLOv5s, resulting in a remarkable mAP of 93.1% for vegetable disease detection. Bora et al. (2023) [18] proposed a system that achieved disease detection rates of 99.84%, 95.2%, 96.8%, and 93.6% for tomato leaves, stems, fruits, and root positions, respectively. Zhang et al. (2023) [19] presented experimental results on 3123 tomato leaf images, comprising 1850 camera-captured images and 1273 obtained from the internet, demonstrating that the proposed M-AORANet achieved a recognition accuracy of 96.47%. Sunil et al. (2023) [20] applied a Multi-Feature Fusion module (MFFN) to classify a publicly available tomato disease dataset, attaining training, validation, and external testing accuracies of 99.88%, 99.88%, and 99.83%, respectively. While the studies mentioned above predominantly used samples with simple backgrounds, their adaptability to complex backgrounds is still limited. Presently, datasets for agricultural disease detection models are predominantly classified into two categories (Fig. 1): those captured in natural environments, featuring backgrounds, and those obtained under controlled conditions, devoid of backgrounds. As depicted in Fig. 1, images obtained from natural environments exhibit intricate backgrounds, contributing to models with enhanced robustness and generalization. In contrast, images acquired in controlled environments exhibit minimal background interference, potentially yielding models that are less effective in natural settings. Only a few studies have addressed disease identification in complex environments, but their network accuracy has not met the desired expectations, indicating certain limitations.

Compared to traditional methods of plant disease detection, deep learning has shown significant advantages in the field [21]. However, the effectiveness of deep learning models largely depends on the training dataset [22]. Due to the significant variability of different diseases in real-world environments, it is challenging for training datasets to cover all scenarios, leading to insufficient sample quantities and imbalanced distributions [23]. Additionally, there is difficulty in establishing unified standards for annotating disease training datasets, which can result in misclassifications when using end-to-end prediction methods like deep learning. Considering that plant diseases often occur alongside various contextual information, utilizing multiple sources of information during disease occurrences can aid in more accurate category judgments. Wang et al. (2020) [24] proposed a context-aware attention network that encodes various types of contextual information into image annotations. Zhao et al. (2020) [25] developed a deep learning system called the Multi-Context Fusion Network (MCFN), which utilizes contextual features collected from image-capturing sensors as prior information to assist in crop disease classification. They reduced false alarms in the proposed ContextNet and designed a deep fully connected network to fuse visual and contextual features for crop disease prediction. Zhou et al. (2021) [26, 27] proposed a model that explores semantic embeddings of disease images and disease description text, integrating the correlation and complementarity between the two modal data types. The region proposal component guides the model to focus on regions of interest in disease images with complex backgrounds in a weakly supervised manner, thereby avoiding the costly manual annotation of key image regions. The progressive learning network allows the model to progressively learn global features and fine local features. Wang et al. (2021) [28] combined modal information from disease image and disease text, achieving better results on small datasets compared to using image or text models alone. Feng et al. (2022) [29] proposed an end-to-end disease recognition model composed of a lesion region detector and a disease classifier (YOLOv5s + BiCMT), demonstrating that bidirectional cross-modal feature fusion of disease images and text is an effective method for in-field vegetable disease identification, with robust performance. Cheng et al. (2023) [30] introduced a location attention block, which effectively extracts positional information from feature maps and constructs attention maps to enhance the model’s feature extraction capabilities in regions of interest. It is evident that integrating deep learning models with multi-source information on plant diseases can lead to a unified solution, thereby improving the accuracy of plant disease detection.

**Fig. 2:**
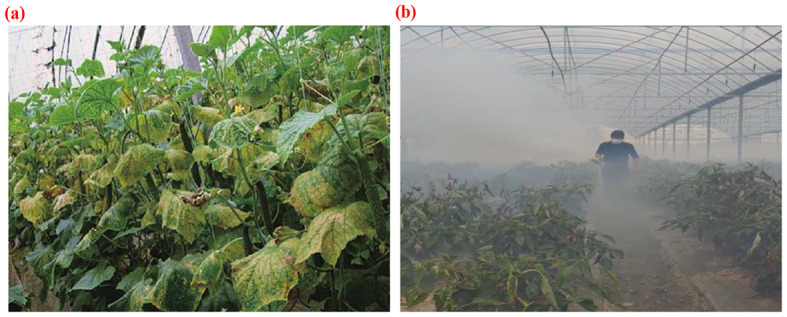
Vegetable disease damage. (**a**) Widespread dissemination of diseases (**b**) Extensive spraying of agricultural chemicals

The study was conducted in Shouguang City, located in Shandong Province, China. Known as the “hometown of vegetables” in China, Shouguang City is renowned for pioneering winter warm greenhouses in the country. It serves as the largest production base for greenhouse vegetables in China, cultivating a diverse range of vegetables, including tomatoes, cucumbers, bitter gourds, eggplants, and chili peppers. These crops, along with eggplants, melons, and legumes, are integral to China’s “vegetable basket project” and are susceptible to various diseases. The greenhouse environment further increases the risk of disease incidence [31]. In recent years, the escalating probability of vegetable diseases due to climate change has necessitated widespread pesticide spraying (Fig. 2). Consequently, residual pesticide levels in vegetables have remained persistently high, posing significant food safety concerns and substantially diminishing the economic viability of vegetable cultivation [32].

**Fig. 3:**
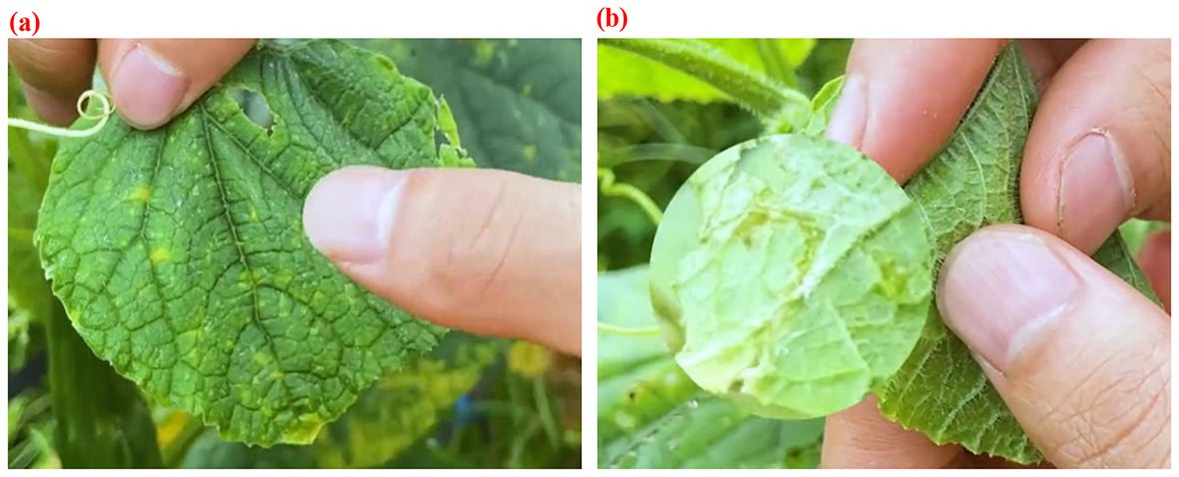
Diagnosis of vegetable diseases using empirical methods. (**a**) Diseases on the front surface of leaves (**b**) Diseases on the back surface of leaves

Therefore, accurate diagnosis of vegetable diseases becomes crucial for effective disease prevention and control. During preliminary visits and research conducted by our project team among vegetable farmers in Shouguang City, the diagnosis and prevention of vegetable diseases primarily rely on experience urrently (Fig. 3), with low timeliness, poor accuracy, and a high demand for personnel with specialized skills, often leading to misdiagnosis and missed detections [33]. A common vision emerged - the desire to utilize modern technology for precise diagnosis of vegetable diseases. This scientific problem addresses the application of machine vision methods to intelligently and accurately detect and recognize vegetable diseases in the greenhouse planting environment.

The detection and identification of vegetable diseases play a crucial role in enabling growers to promptly address them and minimize losses [34]. However, machine vision-based detection and recognition of vegetable diseases encounter significant challenges in real-world planting environments. These challenges include complex planting conditions, various types of diseases, and subtle differences in symptom manifestation. While deep learning technology has made progress in addressing these issues in recent years, improving the accuracy of vegetable disease detection and recognition to meet the requirements of diverse regions, spaces, and timeframes in greenhouse planting remains a key concern.

This study aims to tackle the limitations of insufficient data volume for vegetable disease images in greenhouse planting environments and the subpar detection performance observed in existing research. To achieve this, we construct a large sample dataset comprising disease images captured in greenhouse planting environments. Additionally, we investigate Space-Time Fusion Attention Network and multi-layer encoding and decoding feature fusion networks. Through this project, we develop a vegetable disease detection method that leverages multi-source information fusion. The performance of the suggested approach is assessed using a dataset created specifically for this research. It is anticipated that this project will make significant strides in the crucial area of image recognition for greenhouse vegetable diseases, thereby providing a scientific theoretical foundation for comprehensive disease prevention and control measures.

## Materials and methods

### Materials

#### Data collection

**Fig. 4:**
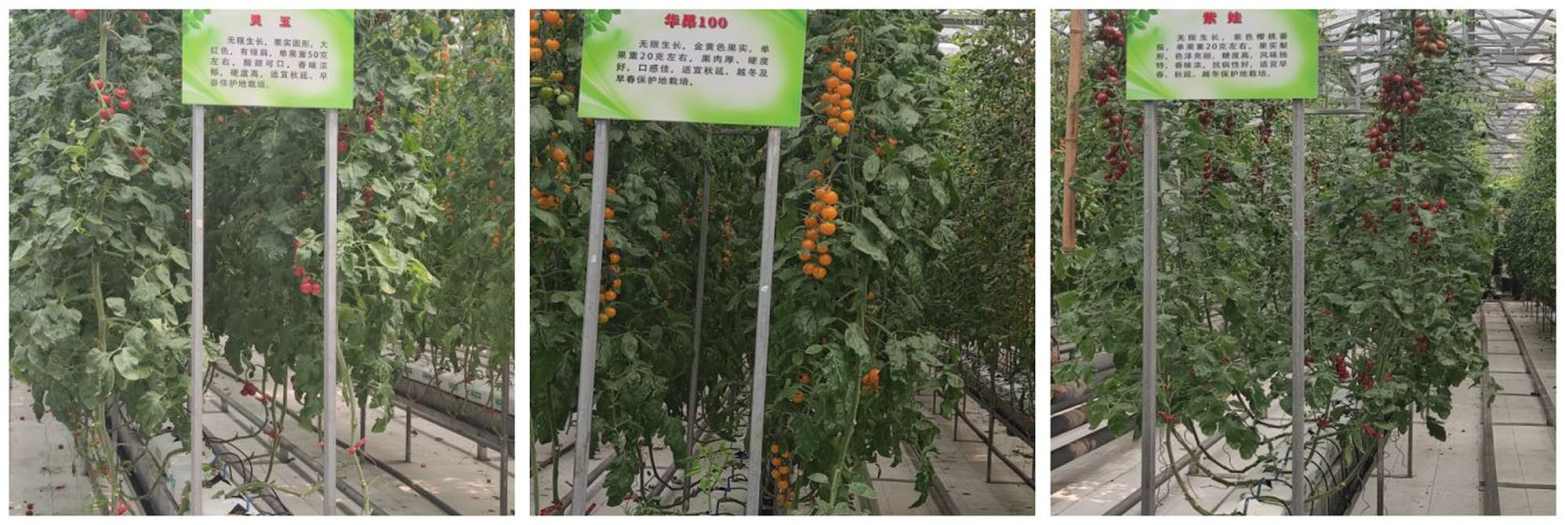
The Vegetable Planting Base

The collection of image data was collaboratively conducted by multiple researchers and agricultural experts from our team. The data collection took place at the Vegetable Planting Base located in Shouguang City, Shandong Province, China (coordinates: 118.782956 E, 36.930686 N). The base encompasses a total area of 680,000 acres and cultivates various types of vegetables, including tomatoes, cucumbers, and bitter melons. Figure 4 illustrates the data collection environment at the base.

**Fig. 5:**
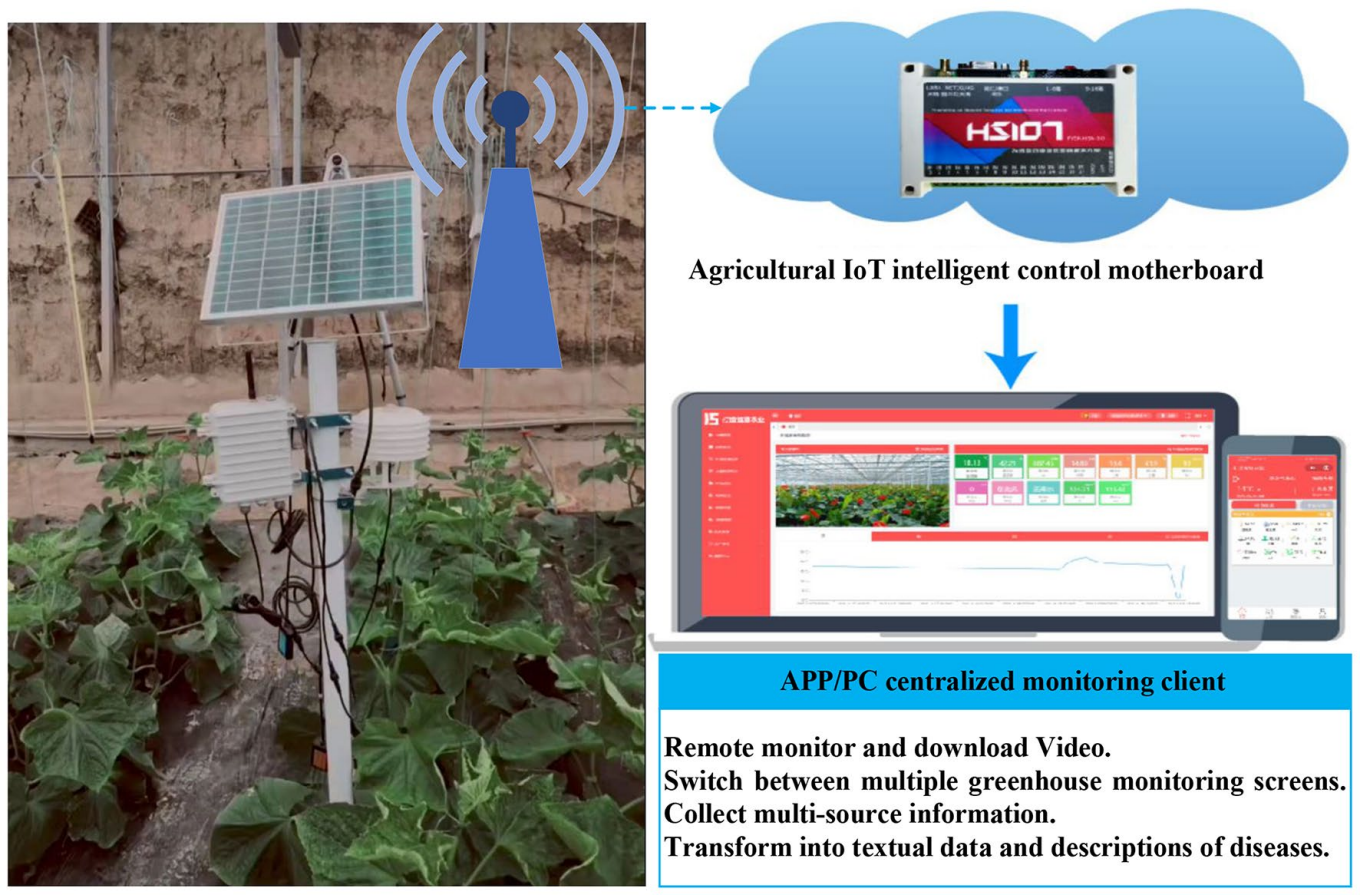
The Internet of Things (IoT) used for Data Collection

At various times, under diverse weather conditions, temperatures, lighting, and angles, images of naturally occurring diseases were captured using greenhouse monitoring cameras. Building upon a thorough analysis of the disease pathogenesis, a large-scale dataset (VDGE, Vegetable Disease for Greenhouse Environment) was established. The images in VDGE are formatted as JPEG. Concurrently, leveraging Internet of Things (IoT) technology, with appropriate sensor support, automatic collection of space, temporal, and environmental information, among other multi-source data, was facilitated (Fig. 5).

The collected multi-source information was then automatically transformed into textual data, providing descriptions of the diseases. The backgrounds of the disease images encompass various noises and environmental factors, such as leaves, weeds, soil, and diverse lighting conditions, rendering them suitable for real-world model applications and offering credible experimental data for deep learning modeling. Examples of collected data are shown in Table 1.

**Table 1:**
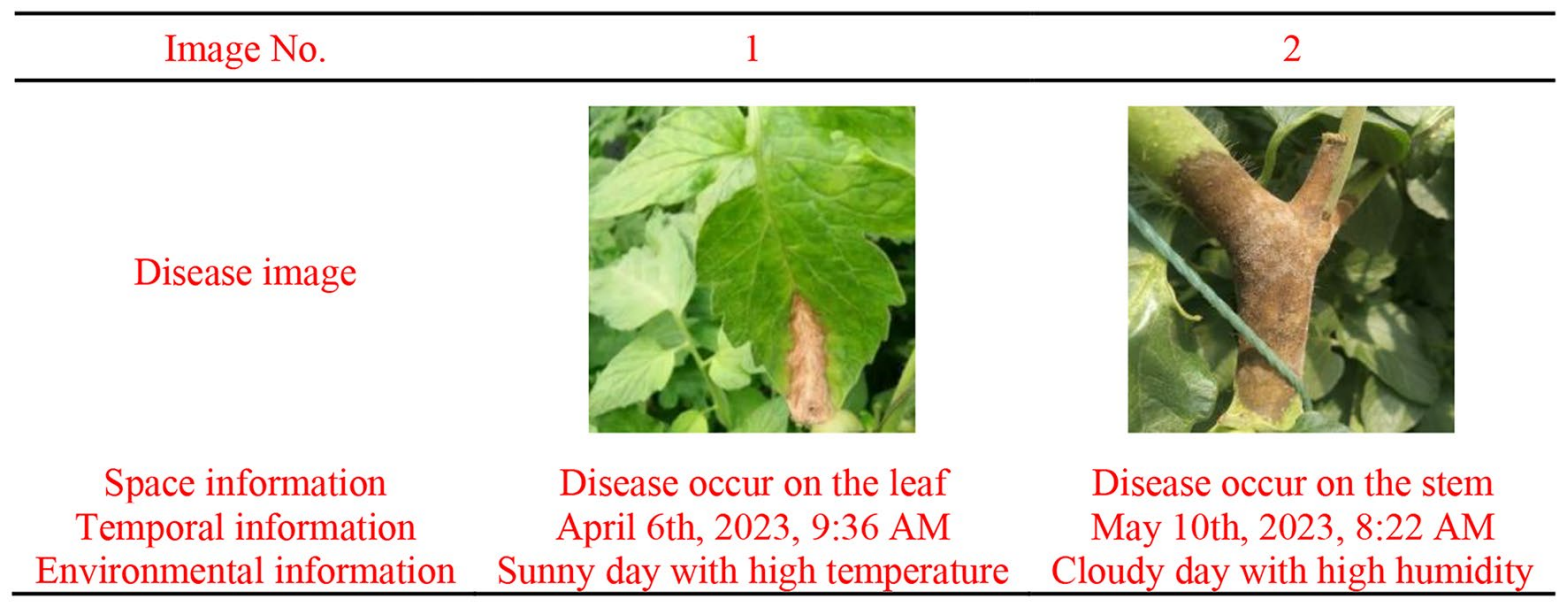
Examples of collected data

#### Data preprocessing

In the initial image collection, certain images were found to be redundant, blurry, or poorly lit. Consequently, a selection process was undertaken to eliminate low-quality images and retain only those with clarity. Moreover, given the surplus of background information in the images, with disease spots occupying only a fraction of the entire image, manual cropping was necessary. This was done to reduce the data volume for more efficient processing and to minimize interference from non-essential elements. All images were resized to a standardized dimension of 640 × 640. Consequently, we obtained a refined original dataset for further research.

#### Data annotation

Employing a semi-supervised approach, disease targets within VDGE were labeled. Initially, a small subset of disease data (1000 images) underwent manual annotation, including disease category labels, infected area location information, bounding boxes delineating regions of interest, and a textual description file containing multi-source information. Subsequently, a model trained on these annotated disease data assigned temporary pseudo-labels to the remaining disease data. These temporary labels were then manually adjusted for certain diseases, followed by continued training of the disease detection model. Through iterative refinement, the performance of the labeling model was enhanced, resulting in savings of manpower and cost compared to complete manual labeling.

During the manual labeling process, to ensure accuracy and authority, images were initially classified into their corresponding high-level vegetable categories, such as tomatoes, cucumbers, and bitter gourds. Subsequently, three groups of agricultural experts independently annotated different types of vegetable diseases and multi-source information texts. Thus, each data received three labels for both the image and multi-source information text. Considering potential inconsistencies among annotations from different annotators, the following measures were taken: data with completely inconsistent labels among the three were removed, while data with two consistent labels or all three consistent labels were retained, with the majority-consistent label chosen as the final label. Additionally, data expressing completely opposite features in images and multi-source information texts were discarded. Statistics on the volume of the original dataset VDGE after data annotation are shown in Table 2.

**Table 2:** Statistics on the volume of the original dataset VDGE after data annotation

Vegetable types	Disease type	Number of images	Number of annotations
Tomato	Early blight	1162	3498
	Late blight	956	2924
	Gray mold	981	3031
	Health	1369	4153
Cucumber	Downy mildew	937	2812
	Gray mold	922	2854
	Viral disease Health	681	2129
		1031	3192
Bitter melon	Downy mildew	698	2193
	Powdery mildew	767	2354
	Viral disease	892	2732
	Health	1168	3576

By analyzing Table 2, it becomes apparent that the dataset has certain issues. These include a limited total sample size of image data, an uneven distribution of samples across different vegetables, and variations in the number of samples for different diseases within the same vegetable category. Consequently, this study aims to mitigate the impact of these challenges on model performance by employing techniques such as data augmentation, model structure optimization, and other related methods.

#### Data augmentation

Data augmentation strategies are pivotal in enriching experimental data, facilitating more realistic simulations of intricate object detection scenarios, and enhancing the performance of detection models. To maintain independence between training and test set images throughout the experimental process and bolster the model’s generalization capability, the dataset was initially partitioned into training, validation, and test sets using an 8:1:1 ratio before any data augmentation operations were conducted.

**Fig. 6:**
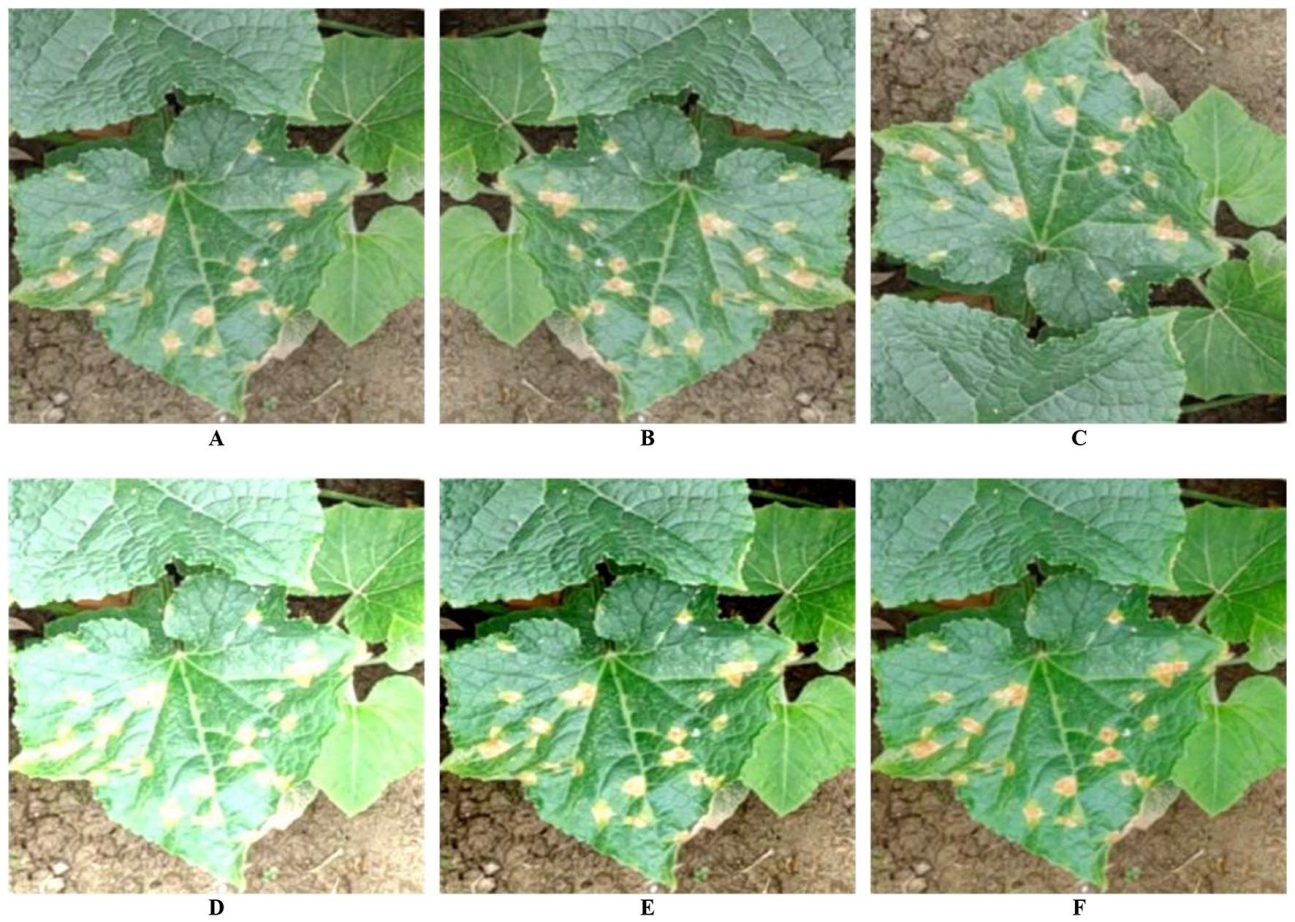
Data Augmentation Effect

In vegetable disease detection scenarios, data augmentation methods such as random cropping, color transformation, and scaling can alter the shape, color, and texture features of the diseases. Therefore, this study employs five specific methods for data augmentation to ensure controlled changes: horizontal flipping, vertical flipping, brightness transformation, contrast transformation, and saturation transformation. The aim is to enhance the randomness of data during model training without excessive augmentation. Different data augmentation methods are randomly combined during the training process, with their respective usage probabilities outlined in Table 3. Figure 6 illustrates the enhancement effect, where A represents the original vegetable disease image, and B, C, D, E, and F showcase the results after applying the five data augmentation operations.

**Table 3:** Probability of Data Augmentation methods

No.	Method	Probability
1	Vertical Flipping	20%
2	Horizontal Flipping	30%
3	Brightness Transformation	15%
4	Contrast Transformation	15%
5	Saturation Transformation	40%

### Methods

This study presents a novel approach for vegetable disease detection by utilizing a multi-source information fusion method. The proposed method is specifically designed to address the challenges posed by complex unstructured environments commonly found in greenhouse settings, including variations in lighting conditions, occlusion, and overlap. To facilitate this research, a dedicated dataset of vegetable disease images grown in greenhouses was constructed. By leveraging this dataset, the developed method demonstrates enhanced adaptability and robustness in accurately detecting and identifying vegetable diseases.

#### Space-Time Fusion attention network (STFAN)

Considering the varying types of diseases that affect different vegetables, it is important to account for factors such as the occurrence time, surrounding environment, and spaceal conditions specific to each type of vegetable disease. Therefore, integrating space, temporal and environmental information from vegetable disease images becomes crucial for accurate disease detection. One approach involves initially classifying vegetables based on multi-source information and subsequently developing disease detection models tailored to each vegetable type. This two-step process allows for a more effective and specialized detection approach, thereby improving accuracy and reliability in identifying and combating vegetable diseases.

**Fig. 7:**
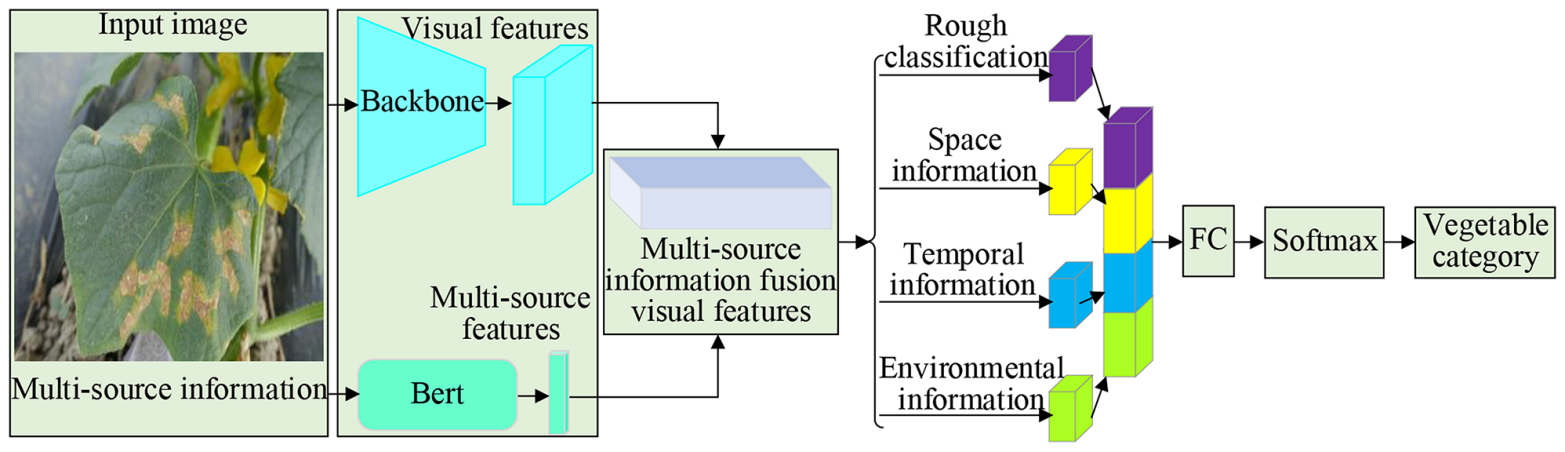
Space-Time Fusion Attention Network (STFAN)

The Space-Time Fusion Attention Network proposed in this study is depicted in Fig. 7. The network takes into account both efficiency and accuracy by utilizing a backbone network to extract multi-source information from the original image. This extracted information is subsequently input into two fully connected layers, forming the decision layer that outputs the specific vegetable type corresponding to the disease image. This approach enables effective utilization of the multi-source information obtained from the image, allowing accurate separation of different types of vegetable data. In Fig. 6, the first branch focuses on generating the coarse classification results for the image. The remaining branches individually output space, temporal, and environmental information. These branches extract specific features related to these aspects from the input image. By concatenating the multi-source information from each branch, and feeding it into a decision network comprising multi-layer perceptrons, the final vegetable classification result can be obtained.

Firstly, following the network structure outlined in Fig. 6, the process commences with the input of vegetable disease images and multi-source information. By utilizing the Bert model [35] to encode space, temporal and environmental information related to vegetable diseases (referred to as multi-source information), a feature matrix of multi-source information is obtained. The textual information of vegetable disease multi-source information is fed into the model, resulting in an output of encoded vectors for multi-source information.

Secondly, the core concept of multi-source information fusion is achieved through attention mechanisms to integrate visual and multi-source information. This involves merging the encoded knowledge features with the visual features corresponding to the input image, thereby obtaining multi-source information fusion visual features.

Thirdly, the network proceeds to extract multi-source information features. This extraction process is facilitated by various branches, each dedicated to capturing distinct types of information such as space, temporal, and environmental features. Once the multi-source information is obtained from these branches, it undergoes fusion through fully connected layers. These layers amalgamate the extracted features, facilitating the integration and combination of diverse information sources. The outcome of this fusion process yields comprehensive and enriched feature representations that encompass the various aspects of the vegetable disease images.

Finally, a softmax classifier is employed to classify and determine the vegetable type based on the fused features. The softmax classifier assigns probabilities to each potential vegetable category, indicating the likelihood of the input image belonging to a specific vegetable type. The output with the highest probability denotes the predicted vegetable type associated with the input disease image.

#### Multilayer encoder-decoder feature Fusion Network (MEDFFN)

After determining the types of vegetables in a vegetable disease image, it becomes necessary to identify the location of the disease. However, most convolutional neural network models reduce the resolution of feature maps in deep convolutional layers to 1/32 or 1/64 of the original image’s size. As a result, small targets like diseases become indiscernible on these deep feature maps, where a target of size 32 × 32 or 64 × 64 occupies only a single pixel. Fortunately, we can leverage the environmental information surrounding vegetable diseases as additional multi-source data to aid in disease detection tasks. It is important to note that diseases typically manifest on the vegetables themselves rather than randomly appearing in the sky or elsewhere. Hence, the small-scale multi-source information derived from shallow convolutional blocks, which includes details about the texture, color, shape, and other characteristics of the disease’s surrounding environment, can be integrated with the deep higher-order semantic information to generate super-resolution features. This integration process serves two purposes. Firstly, it prevents the loss of important details in deep features by incorporating the fine-grained information from shallow convolutional blocks. Secondly, it ensures an appropriate receptive field size, enabling accurate disease detection.

**Fig. 8:**
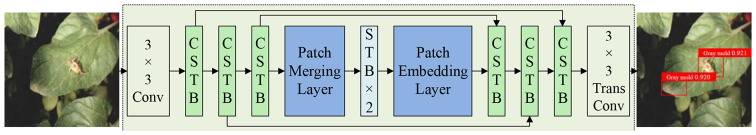
Multilayer Encoder-Decoder Feature Fusion Network (MEDFFN)

This study introduces a multi-layer encoding and decoding feature fusion network, illustrated in Fig. 8. A novel module called CSTB (Convolutional Swin Transformer Block) is proposed, which combines convolutional modules with the Swin Transformer architecture. This module is utilized to construct a multi-layer encoding and decoding feature fusion network. To enhance the feature information of interest and suppress redundant information, the encoder incorporates an upsampling layer, while the decoder includes a downsampling layer. These layers work together to provide improved local disease feature details for the encoding and decoding sequence features. By leveraging this approach, the network can effectively fuse and integrate information from multiple layers, leading to enhanced performance in disease object detection.

**Fig. 9:**
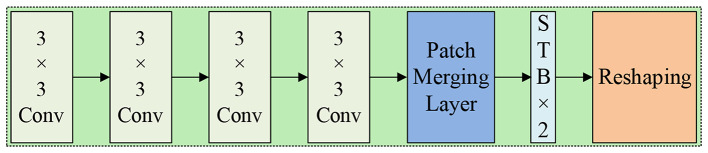
Structure of Convolutional Swin Transformer Block (CSTB)

As depicted in Fig. 9, the hybrid model, Convolutional Swin Transformer Block (CSTB), consists of multi-layer convolutions and Swin Transformer Block (STB). The encoding process utilizes a patch merging layer, while the decoding process employs a patch embedding layer. The input CSTB undergoes a feature forward pass determined by a 1 × 1 convolution, followed by three stacked convolutional modules. This module facilitates channel dimension operations, both upsampling and downsampling, while preserving the spatial dimension in the output. It effectively integrates information across channels. By employing multi-layer convolution modules, the model can learn complex and abstract feature information, extract more detailed spatial features, and provide STB with structural priors. Subsequently, the output passes through either the patch merging layer or the patch embedding layer in the forward direction. The patch merging layer is utilized for downsampling in the encoding process, while the patch embedding layer is employed for upsampling during the decoding process.

**Fig. 10:**
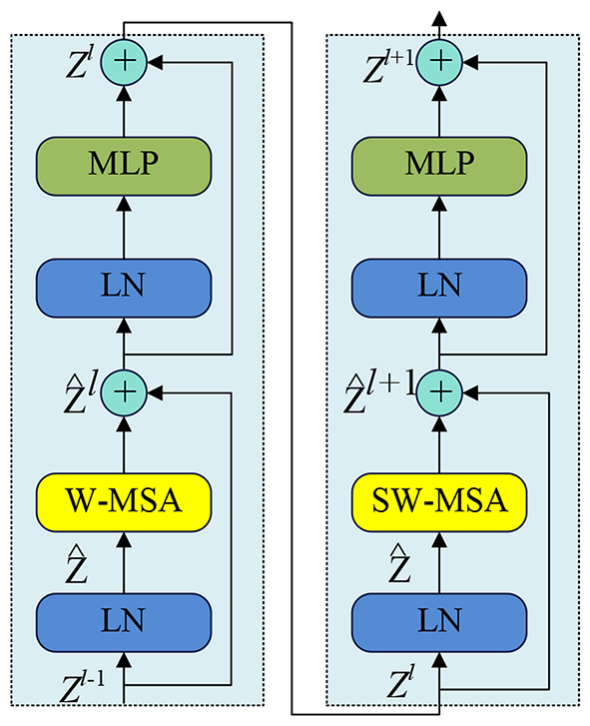
Structure of two successive Swin Transformer blocks (STB)

Once the encoding has been downsampled using the patch merging layer or the decoding has been upsampled through the patch embedding layer, it is then forwarded into the two-layer Swin Transformer Block (STB), as depicted in Fig. 10. This module comprises two stacked layers of Swin Transformer Layers (STL). Each STL is composed of layer normalization, a local window multi-head attention module (W-MSA), residual connections, and linear layers. Specifically, two consecutive Swin Transformer Layers utilize a multi-head self-attention module based on local windows. The calculation formula for two consecutive Swin Transformer Layers can be expressed as follows:

1 $${\widehat{Z}}^{l} = WMSA\left(LN\left({Z}^{l-1}\right)\right)+{Z}^{l-1}$$

2 $${z}^{l}=W\left(LN\left({\widehat{Z}}^{l}\right)\right)+{\widehat{Z}}^{l}$$

3 $${\widehat{Z}}^{l+1} = WMSA\left(LN\left({Z}^{l}\right)\right)+{Z}^{l}$$

4 $${Z}^{l+1} = W\left(LN\left({\widehat{Z}}^{l+1}\right)\right)+{\widehat{Z}}^{l+1}$$

In the equation, ${\widehat{Z}}^{l}$ and ${z}^{l}$ represent the output of the local window multi-head attention module and linear layer in the first STL layer, respectively. Similarly, ${\widehat{Z}}^{l+1}$ and ${Z}^{l+1}$ represent the output of the local window multi-head self-attention module and linear layer in the second STL layer, respectively. The function WMSA(·) denotes the local window multi-head attention operation, LN represents layer normalization, and W(·) represents the linear layer.

Unlike conventional Transformer, this model employs a multi-head self-attention mechanism within non-overlapping local windows. This approach enhances the extraction of local feature information. For the local window multi-head self-attention, let’s consider a 2D feature map $X?{R}^{C\times H\times W}$, where H and W denote the vertical and horizontal dimensions of the feature map correspondingly. To implement this, the feature map is split into non-overlapping windows of size M × M. Then, we flatten and transpose each window to obtain the feature ${X}^{i}?{R}^{{M}^{2}\times C}$. Subsequently, we apply multi-head self-attention operations to the flattened features within each window. Assuming the number of heads is k, and the feature dimension of a single head is ${d}_{k}=C/k$, the formula for calculating the k-th multi-head self-attention within a non-overlapping window is as follows:

5 $${Y}_{k}^{i} = Attention\left({X}^{i}{W}_{i}^{q},{X}^{i}{W}_{i}^{k},{X}^{i}{W}_{i}^{v}\right), i=1,\cdots ,N$$

In the equation, ${W}_{i}^{q},{W}_{i}^{k},{W}_{i}^{v}$ represent the query, key, and value weight matrices for the k-th multi-head self-attention, respectively. ${Y}_{k}^{i}$ represents the output of the k-th multi-head self-attention operation, which is then concatenated and layer normalized to obtain the final output, denoted as ${\widehat{X}}_{k}$. The calculation formula can be expressed as follows:

6 $${\widehat{X}}_{k}= LN\left(Concat\left({Y}_{k}^{1},{Y}_{k}^{2},{\cdots ,Y}_{k}^{M}\right)\right)$$

In the formula, Concat(·) represents the concatenation operation, and LN represents layer normalization.

After passing through the two STB modules, the features are then forwarded to the next stage, which is the CSTB module. In this module, the features need to be reshaped in order to restore the required image feature dimensions before being input into the convolutional layer of the next CSTB stage. Specifically, the output sequence dimensions of the second STB are (1, H × W, C). The reshaping process involves restoring the image feature dimensions to (C, H, W) before feeding them into the convolutional layer of the subsequent CSTB stage.

#### Boundary structure loss function (BSLF)

To address the challenges of missed and false detections in small-scale disease detection, a boundary structure loss function (BSLF) is introduced. This loss combines the boundary intersection over union ratio loss (IOU) [35], structural similarity loss (SSIM) [36], and the widely used cross-entropy loss (BCE) [37].

The following equation represents the calculation formula for the loss function:

7 $${L}_{fusion}= {\lambda }_{1}{L}_{bce}+{\lambda }_{2}{L}_{iou}+{\lambda }_{3}{L}_{ssim}$$

In the equation above, ${L}_{bce}, {L}_{iou}, {L}_{ssim}$ represent the cross-entropy loss, boundary intersection over union ratio loss, and structural similarity loss, respectively. ${\lambda }_{1}, {\lambda }_{2}, {\lambda }_{3}$ are weight coefficients, which are set to 1.

The cross-entropy loss is commonly used in binary classification or segmentation tasks to measure the discrepancy between the predicted values and the ground truth labels. It is described by the following equation:

8 $${L}_{bce}= \sum _{x=1}^{H} \sum _{y=1}^{W}\left[{G}_{\left(x,y\right)}\text{log}{S}_{\left(x,y\right)}+\left(1-{G}_{\left(x,y\right)}\right)\text{log}\left(1-\text{log}{S}_{\left(x,y\right)}\right)\right]$$

In the equation above, ${G}_{\left(x,y\right)}?\left(\text{0,1}\right)$ represents whether the pixels at coordinates $\left(x,y\right)$ belong to the object in the ground truth labels of vegetable diseases. ${S}_{\left(x,y\right)}$ represents the predicted probability that the pixels at coordinates $\left(x,y\right)$ belong to the disease target.

Due to the challenge of class imbalance commonly encountered in disease detection, cross-entropy loss, which estimates overall classification accuracy for all pixels equally, may not effectively handle this issue. To address this, the boundary intersection over union (IOU) ratio loss is introduced to penalize inaccurate classification and improve regional consistency and boundary response. The IOU loss is defined as follows:

9 $${L}_{iou}= 1-\frac{\sum _{x=1}^{H} \sum _{y=1}^{W}{S}_{\left(x,y\right)}{G}_{\left(x,y\right)}}{\sum _{h=1}^{H} \sum _{w=1}^{W}\left({G}_{\left(x,y\right)}+{S}_{\left(x,y\right)}-{S}_{\left(x,y\right)}{G}_{\left(x,y\right)}\right)}$$

Similar to the cross-entropy loss, ${G}_{\left(x,y\right)}?\left(\text{0,1}\right)$ denotes whether the pixels at coordinates $\left(x,y\right)$ belong to the object in the ground truth values of vegetable diseases. ${S}_{\left(x,y\right)}$ represents the predicted probability that the pixels at coordinates $\left(x,y\right)$ belong to the disease target.

Structural similarity loss, originally introduced for image quality assessment, is utilized to capture the structural characteristics within an image. In this context, it is incorporated into the training loss to guide the network in learning the structural information of defective objects based on the provided labels. Let $x=\left\{{x}_{j}:j=1,\cdots ,{N}^{2}\right\}$ and $y=\left\{{y}_{j}:j=1,\cdots ,{N}^{2}\right\}$ represent the pixel values of the N×N feature map for the predicted probability S and the corresponding real label G, respectively. The loss associated with structural similarity can be defined as follows:

10 $${L}_{ssim}= 1-\frac{\left(2{\mu }_{x}{\mu }_{y}+{C}_{1}\right)\left(2{\sigma }_{xy}+{C}_{2}\right)}{\left({\mu }_{x}^{2}+{{\mu }_{y}^{2}+C}_{1}\right)\left({\sigma }_{x}^{2}+{\sigma }_{y}^{2}+{C}_{2}\right)}$$

In the given equation, ${\mu }_{x}, {\mu }_{y}$ and ${\sigma }_{x}, {\sigma }_{y}$represent the mean and standard deviation of x and y, respectively. ${\sigma }_{xy}$ denotes the covariance between x and y. ${C}_{1}$ is assigned a value of 0.012, and ${ C}_{2}$ is set as 0.032.

#### Multisource Information Fusion Method for Vegetable Disease Detection (MIFV)

**Fig. 11:**
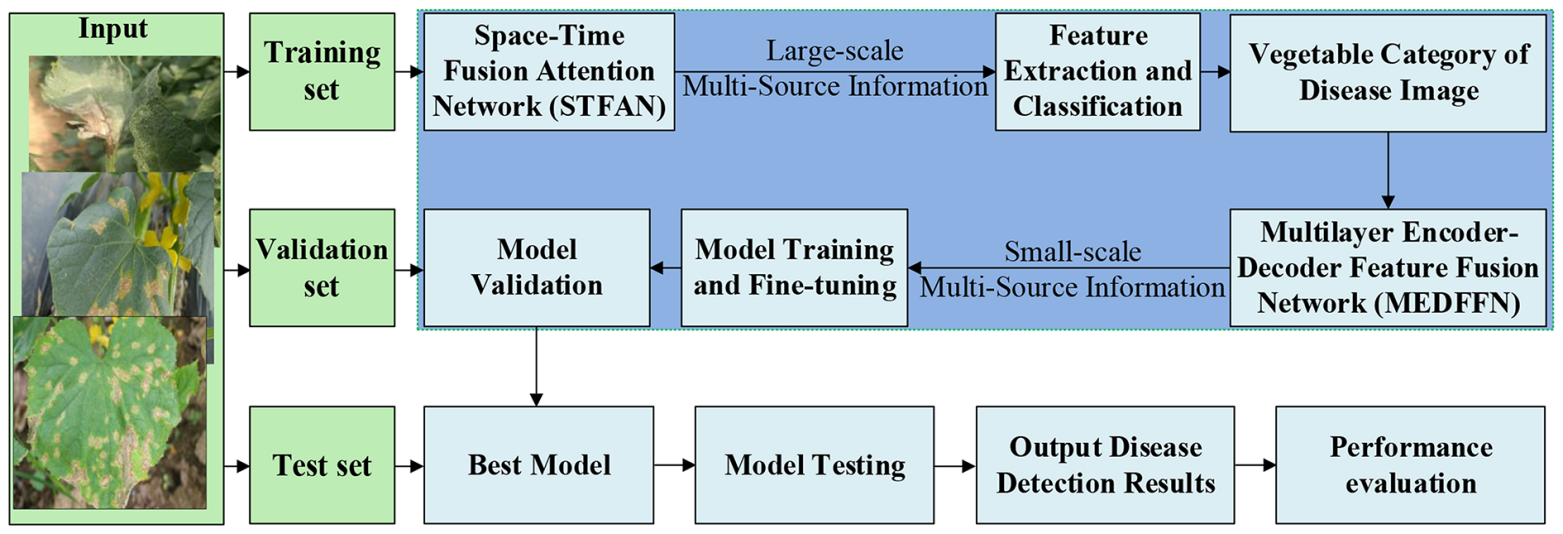
Work flow of Multisource Information Fusion Method for Vegetable Disease Detection

Based on the principles of deep learning, we analyze the relationship between space-time fusion attention network and multi-layer encoding and multilayer encoder-decoder feature fusion network. We explore detection and recognition methods for disease types and positions under challenging conditions like uneven lighting, partial occlusion, and leaf overlap. To further improve disease detection and recognition, a multisource information fusion method for vegetable disease detection (MIFV) method specifically designed for vegetable disease detection (Fig. 11) is introduced.

According to Fig. 11, a multi-layer encoding and decoding feature fusion network is employed for the detection of diseases on specific vegetables. This indicates that there exist significant variations in disease detection models across different vegetables. However, it should be noted that Space-Time Fusion Attention Network do not guarantee the accurate classification of vegetable disease images in every real-world scenario. Consequently, to mitigate the impact of image misclassification and enhance the system’s accuracy and robustness, a pre-training model in the form of a Space-Time Fusion Attention Network is utilized to train a multi-layer encoding and decoding feature fusion network. During the training process, fine-tuning is required for the classification branch of the multi-layer encoding and decoding feature fusion network. In other words, the multi-layer encoding and decoding feature fusion network specific to each type of vegetable needs to undergo a certain number of training iterations using data from other types of vegetables. This step ensures that the Space-Time Fusion Attention Network can still yield accurate detection outcomes even in cases of misclassification.

## Results and discussion

### Experimental environment

In this study, the MIFV model is developed and trained using the Python deep learning framework. The experimental setup involves using an NVIDIA GeForce 3060 Ti graphics card with 32GB of memory. For model training, specific parameters are defined. The batch training size is set to 8, indicating that 8 samples are processed in each iteration. The random gradient descent (SGD) optimizer is employed, with a momentum parameter of 0.937, enabling faster convergence towards optimal solutions. The total epoch count for training is set to 200.

### Evaluating indicator

This study used AP (Average Precision), mean Average Precision (mAP), recall rate (Recall), model parameter (M), computational complexity (GFLOPs) and detection speed (FPS) as evaluation indicators for the target detection model.

The calculation formula for mAP is:

11 $$mAP=\frac{\sum _{i=1}^{k}{AP}_{i}}{K}$$

The formula includes the following elements: K represents the count of detected categories; mAP denotes the mean AP value, with higher values indicating superior detection performance.

AP refers to the AUC (Area Under the Curve) of the PR curve constructed by employing Recall as the x-axis and Precision as the y-axis for a specific category across all predicted images. The calculation formula for Precision, Recall and AP are as follows:

12 $$P=\frac{TP}{TP+FP}$$

13 $$R=\frac{TP}{TP+FN}$$

14 $$AP={\int }_{0}^{1}P\left(r\right)dr$$

In the provided formula, TP signifies the count of detection frames that fulfill the criteria of having an IOU value greater than or equal to the specified threshold. FP, on the contrary, represents the quantity of detection frames with an IOU value lower than the prescribed threshold. FN denotes the number of targets that were not correctly identified.

Frames Per Second (FPS) is a measure of a model’s inference velocity. A model is considered to satisfy real-time detection criteria when it achieves an FPS exceeding 30. Additionally, Giga Floating Point Operations Per Second (GFLOPs) and the model’s parameter volume, are metrics that gauge the model’s computational complexity. Lower values of GFLOPS and parameter volume signify that the model demands less computational resources.

### Selection of learning rate

**Fig. 12:**
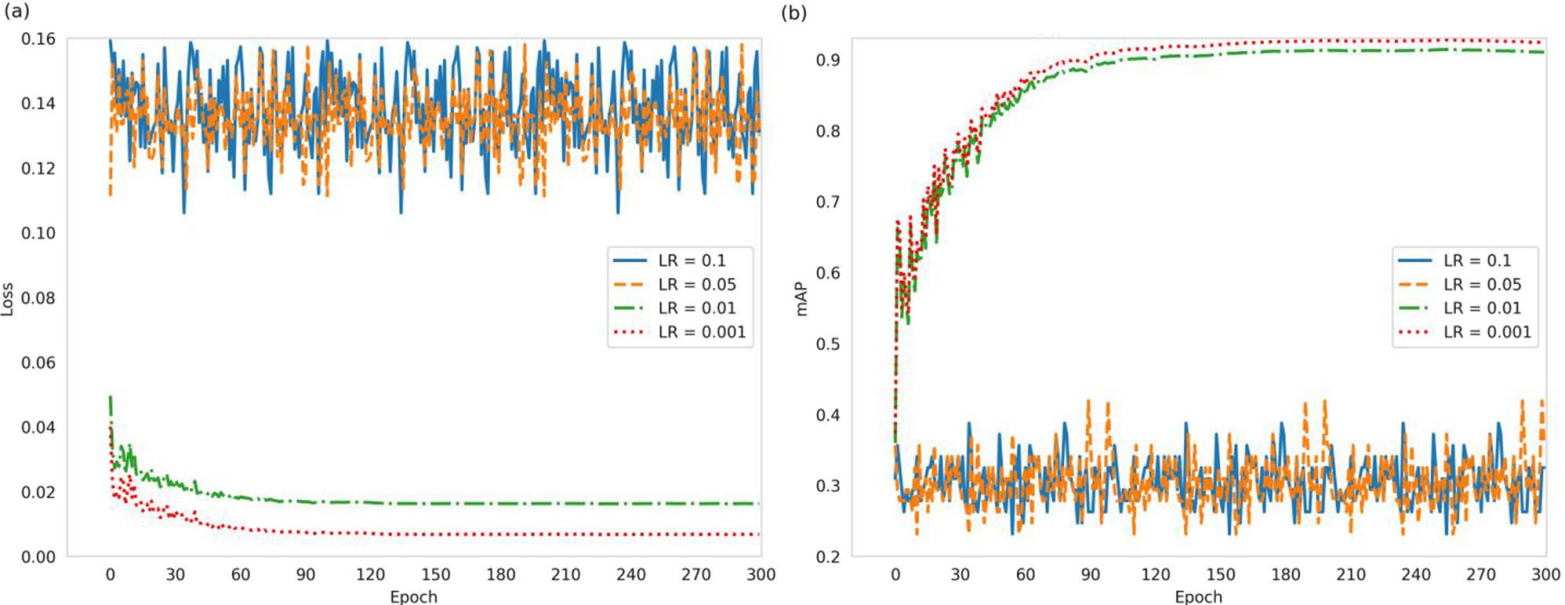
Comparison of the loss function and mAP at different learning rates

The learning rate is a pivotal hyperparameter that significantly influences a model’s performance. This section delves into the optimization of this parameter by evaluating the model’s behavior across a spectrum of learning rates: 0.1, 0.05, 0.01, and 0.001, under otherwise identical conditions. The objective is to enhance the model’s learning efficacy on the training set and its recognition accuracy on the test set. The variation in the loss function across these learning rates is depicted in Fig. 12(a). It is evident that at learning rates of 0.1 and 0.05, the loss function remains largely unchanged, indicating that the step size is too large for the function to converge. This underscores the necessity of selecting a moderate learning rate that balances convergence and the rate of convergence. A comparison between the 0.01 and 0.001 learning rates reveals that the latter offers superior convergence properties and speed. Additionally, Fig. 12(b) illustrates that the accuracy fluctuates markedly under the 0.1 and 0.05 learning rates. In contrast, the highest model accuracy is achieved with a learning rate of 0.001. Consequently, the learning rate is ultimately set to 0.001.

### Model training

**Fig. 13:**
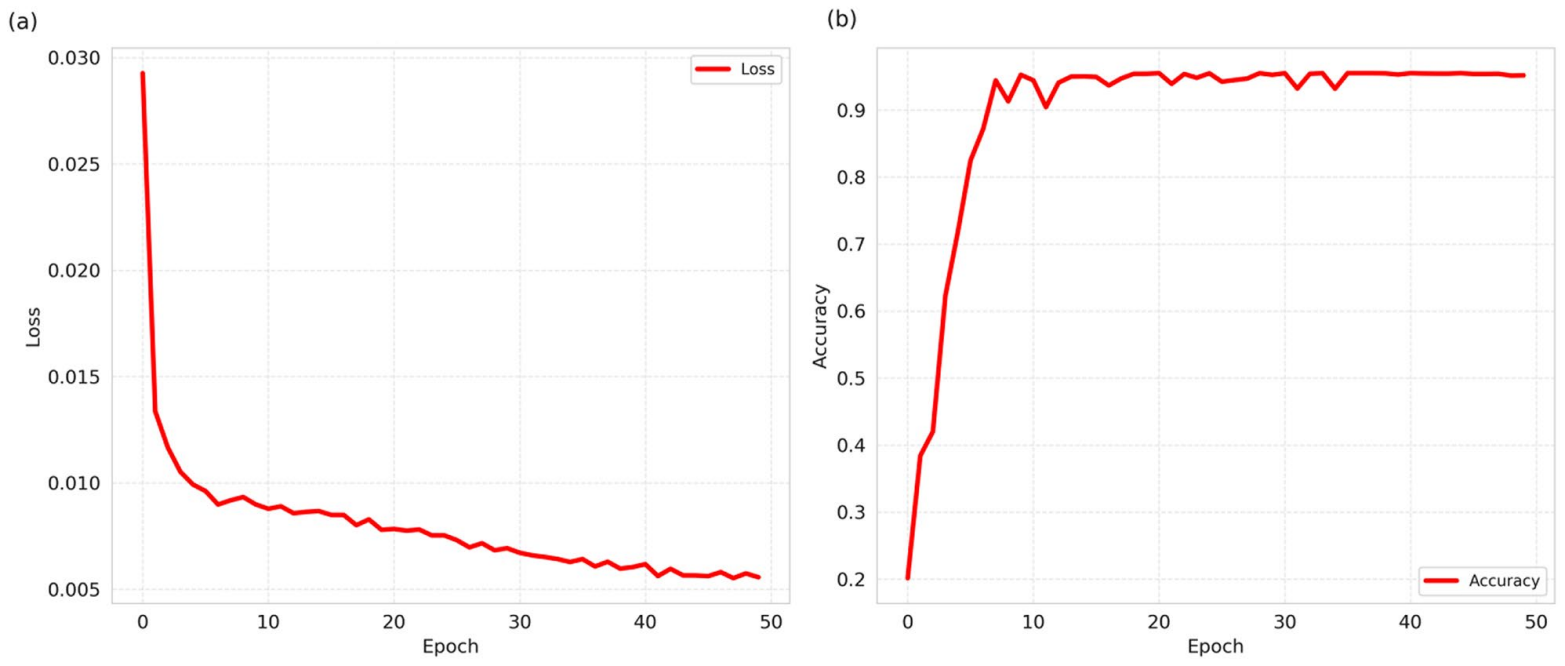
The change curves of the loss function and accuracy during the pretraining phase of STFAN

During the model training process, we employed the STFAN as a pretraining model and utilized the learned features to train the MEDFFN. Initially, during the pretraining phase of STFAN, we observed a continuous decrease in the loss function, accompanied by a steady increase in accuracy, as depicted in Fig. 13. This serves as evidence of the effectiveness of STFAN in feature learning.

**Fig. 14:**
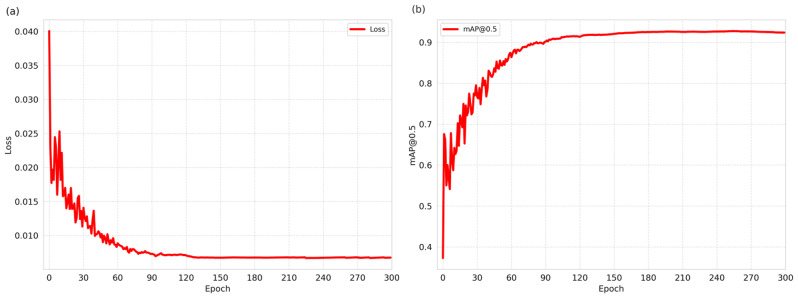
The curves depicting the variation of the loss function and mAP during the fine-tuning phase of MEDFFN

Subsequently, we proceeded to the fine-tuning phase of MEDFFN. During this stage, particular attention was directed towards fine-tuning the classification branch to mitigate the potential impact of misclassifications from STFAN on the final detection results. Specifically, we devised a targeted fine-tuning strategy, wherein each vegetable category’s MEDFFN underwent multiple training sessions, encompassing both intra-category and inter-category vegetable data. This approach aimed to enhance the model’s robustness against misclassifications. Throughout the fine-tuning process, the loss function steadily decreased, accompanied by a gradual improvement in the mAP metric, as illustrated in Fig. 14, vividly demonstrating the enhancement in model performance.

To comprehensively assess the performance of the MIFV algorithm proposed in this study, we conducted tests on the test set. Through the analysis of vegetable disease detection results, the detection and false alarm situations of different types of diseases can be evaluated. Utilizing the proposed MIFV model for the detection of various types of vegetable diseases separately, the precision (P), recall (R), and AP@0.5 (i.e., the AP value when IOU is set to 0.5) are presented in Table 4.

**Table 4:** Results of Vegetable Disease Detection

Vegetable type	Disease type	Precision(%)	Recall(%)	AP@0.5 (%)
Tomato	Early blight	91.6	90.1	90.26
	Late blight	91.4	89.7	90.68
	Gray mold	91.2	89.3	91.33
	Health	99.9	96.3	98.96
Cucumber	Downy mildew	92.2	87.5	89.05
	Gray mold	90.9	86.3	88.32
	Viral disease	93.4	90.8	92.56
	Health	99.8	94.9	98.77
Bitter melon	Downy mildew	90.4	85.8	86.08
	Powdery mildew	87.9	84.8	85.29
	Viral disease	89.2	86.3	87.61
	Health	99.3	93.9	98.52

As shown in Table 4, overall, the proposed MIFV model achieves precision (P), recall (R), and AP values of 85% or higher across 12 types of diseases and healthy samples for three vegetables. It demonstrates a high level of precision and recall in detecting various types of diseases. The mean average precision (mAP) is 92.38%, affirming the MIFV model’s robust capability in detecting vegetable diseases.

### Comparative experiment

This study selected SSD, Faster RCNN, YOLOv5n, YOLOX-s, YOLOv6-N, YOLOv7-tiny, YOLOv8n, and YOLOv9 for comparison using the dataset constructed internally named VDGE. Table 5 illustrates the specific outcomes.

**Table 5:** Comparison of different algorithms

Algorithm	mAP(%)	Model parameter (M)	Computational complexity (GFLOPs)	FPS
SSD (VGG16)	82.09	27.66	63.92	53.6
Faster RCNN (ResNet50)	84.67	140.81	382.38	7.1
YOLOv5n	85.31	47.63	111.36	41.3
YOLOX-s	86.69	55.31	163.04	33.1
YOLOv6-N	87.89	59.92	150.41	35.3
YOLOv7-tiny	88.95	38.73	110.39	42.5
YOLOv8n	89.36	44.85	116.78	40.2
YOLOv9	90.23	42.19	110.23	42.9
MIFV	92.38	39.07	108.92	43.6

Table 5 clearly demonstrates the effectiveness of the MIFV algorithm proposed in this study. On our self-built VDGE dataset, compared to YOLOv7-tiny, YOLOv8n, and YOLOv9, the mAP has been improved by 3.43%, 3.02%, and 2.15% respectively, showcasing significant performance advantages.

It is particularly worth emphasizing that the MIFV algorithm not only excels in detection accuracy but also demonstrates superior performance in terms of model parameters and computational complexity. Compared to existing algorithms, the MIFV algorithm has made significant strides in reducing model parameters and computational complexity, with model parameters at 39.07 M and computational complexity at 108.92 GFLOPs. This is of great importance for devices with limited resources and for application scenarios that require rapid response.

It can be observed from the table that the SSD (VGG16) algorithm has the highest FPS, reaching 53.6, indicating that its inference speed is extremely fast and well exceeds the standard for real-time processing. The Faster RCNN (ResNet50) algorithm has an FPS of 7.1, which is below the standard for real-time processing, suggesting that it may face issues with slow speed in practical applications. The YOLO series of algorithms perform relatively well in terms of FPS, all surpassing the threshold of 30. In particular, YOLOv5n and YOLOv7-tiny have FPS values of 41.3 and 42.5, respectively, indicating that these two algorithms maintain a high mAP while also exhibiting good real-time performance. The MIFV algorithm has an FPS of 43.6, slightly higher than that of YOLOv7-tiny. This indicates that the MIFV algorithm can provide satisfactory inference speed in practical applications, meeting the requirements for real-time detection. Overall, although the MIFV algorithm has the highest mAP, reaching 92.38%, its FPS is also maintained at a high level. This demonstrates that the algorithm ensures high precision while also taking into account inference speed, making it suitable for practical application scenarios.

**Fig. 15:**
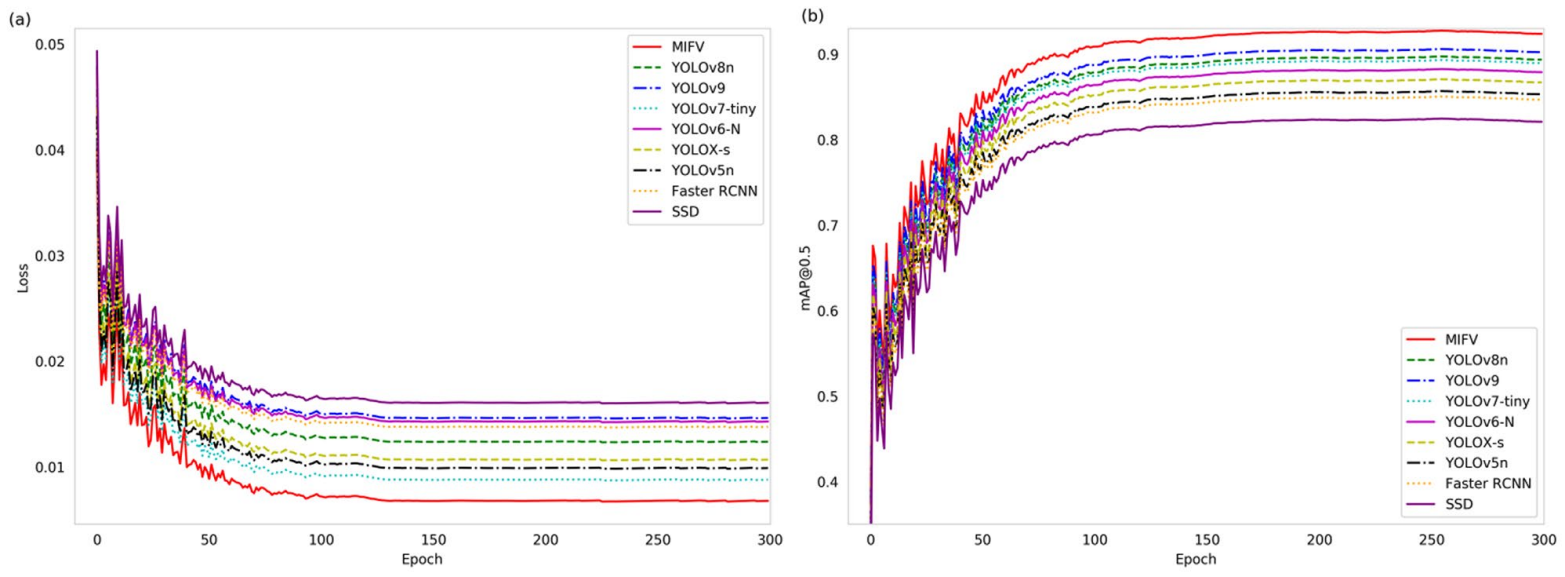
Comparison of Loss and mAP Curves

To substantiate the merits of the proposed MIFV model, this study conducts a comparative analysis with other models, as illustrated in Fig. 15(a) and 15(b), which depict the loss and mAP curves, respectively.

As observed in Fig. 15(a), the proposed MIFV model exhibits a lower loss compared to other models, with an initial loss of 0.040048 that stabilizes around 0.0067. The initial loss is significantly reduced, and the convergence rate is markedly accelerated, demonstrating a smooth curve without pronounced fluctuations. Figure 15(b) reveals that the mAP of the proposed MIFV surpasses that of other models, ultimately converging at approximately 92.38%. This represents a substantial enhancement relative to the mAP values of other models.

**Fig. 16:**
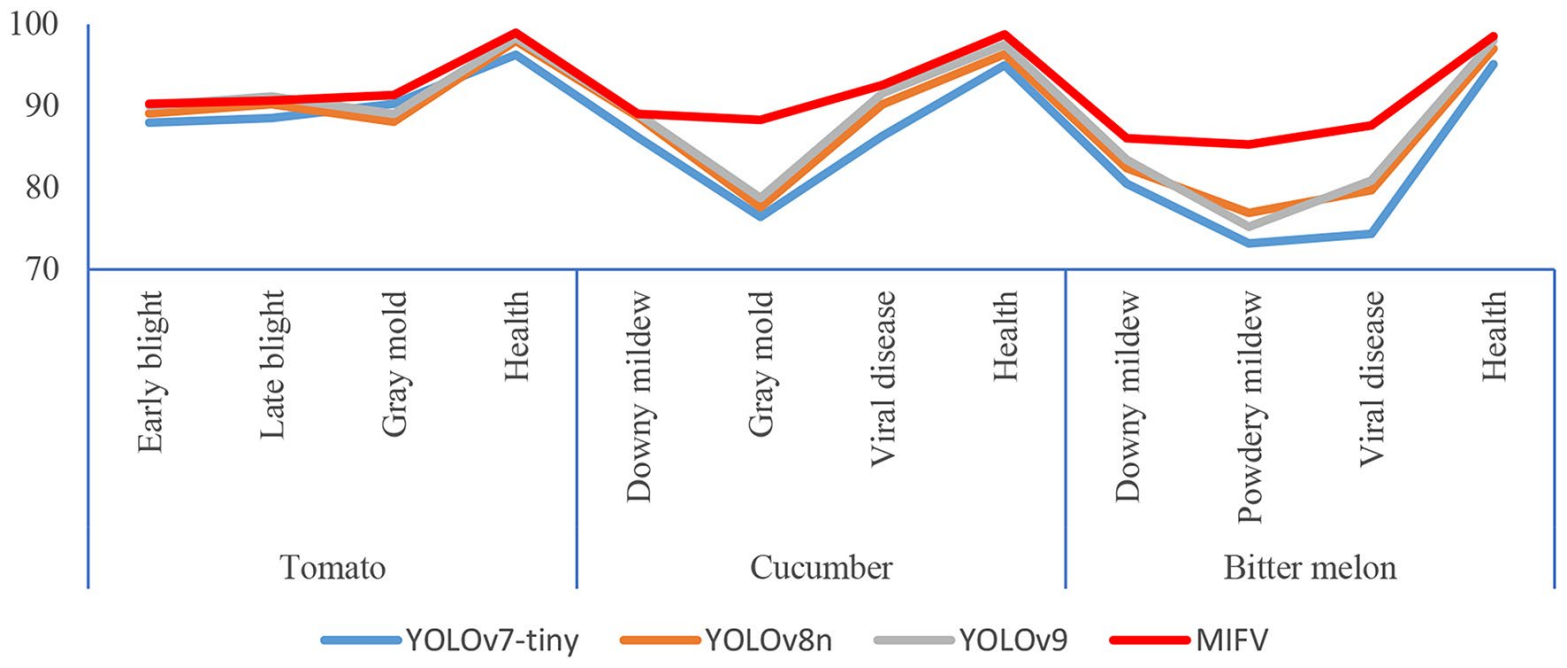
Detection accuracy for different disease categories

To provide a more comprehensive understanding and comparison of the performance of different algorithms, we have detailed in Table 6 the detection accuracies of the YOLOv7-tiny, YOLOv8n, and YOLOv9 algorithms across various target categories, and we have presented an intuitive visualization of these results in Fig. 16. These data and charts offer readers a clear perspective for performance comparison, aiding in a deeper understanding of the advantages of the MIFV algorithm in all aspects. In summary, while maintaining high precision, the MIFV algorithm effectively reduces model parameters and computational complexity, providing strong support for its deployment in practical applications.

**Table 6:** Detection accuracy for different target categories (%)

Vegetable type	Disease type	YOLOv7-tiny	YOLOv8n	YOLOv9	MIFV
Tomato	Early blight	87.96	89.07	90.03	90.26
	Late blight	88.53	90.23	91.17	90.68
	Gray mold	90.26	88.08	89.04	91.33
	Health	96.27	97.99	98.36	98.96
Cucumber	Downy mildew	86.16	88.67	89.02	89.05
	Gray mold	76.45	77.63	78.77	88.32
	Viral disease Health	86.31	90.22	91.59	92.56
		95.01	96.35	97.55	98.77
Bitter melon	Downy mildew	80.51	82.39	83.41	86.08
	Powdery mildew	73.18	76.92	75.22	85.29
	Viral disease	74.37	79.69	80.88	87.61
	Health	95.12	97.03	98.01	98.52

It can be seen that compared to other algorithms, this research algorithm has achieved certain performance improvements for different types of vegetable diseases. The experiment shows that the network design that fully utilizes multi-source information from vegetable disease images is reasonable and improves detection accuracy.

### Ablation experiment

In this study, the MIFV model proposes three significant structural improvements: the STFAN module, MEDFFN module, and BSLF. To validate their effectiveness, ablation experiments were conducted on the VDGE dataset by gradually integrating each module into the Swin Transformer Baseline network, as shown in Table 7.

**Table 7:** Experimental analysis on component elimination

Experiment	Method	mAP(%)	Model parameter (M)
A	Baseline	81.69	38.59
B	A + STFAN	85.96	37.16
C	A + MEDFFN	84.35	39.87
D	C + STFAN	89.08	38.92
E	D + BSLF	92.38	39.07

Based on Table 7, several observations can be made. In Experiment B, the utilization of the STFAN module resulted in a 4.27% increase in mAP, accompanied by a reduction of 1.43 M parameters. This indicates that the STFAN module effectively enhances performance while reducing parameter quantity. Furthermore, in Experiment C, the incorporation of the MEDFFN module led to a 2.66% increase in mAP, with a corresponding increment of 1.28 M parameters. This demonstrates that the MEDFFN module is successful in filtering noise and addressing the issue of disease target feature disappearance in deep convolutional blocks. Building upon the findings of Experiment C, the addition of the STFAN module further improved performance without introducing any additional parameters. The parameter quantity remained consistent with the benchmark model, and the mAP increased by 4.73%. Consequently, the STFAN module effectively supplements multi-source information of vegetable diseases with minimal computational overhead, thereby enhancing the accuracy of vegetable disease target detection. Based on experiment D, the adoption of the improved BSLF loss function resulted in a 3.3% increase in mAP, without introducing any additional parameter quantities. This affirms the effectiveness of the enhanced loss function in vegetable disease target detection. Ultimately, the final improved model exhibited a remarkable improvement of 10.69% in mAP compared to the benchmark model, with only a marginal increase of 0.48 M parameters. These compelling results provide strong evidence for the efficacy of the various modules designed and incorporated in this study.

**Fig. 17:**
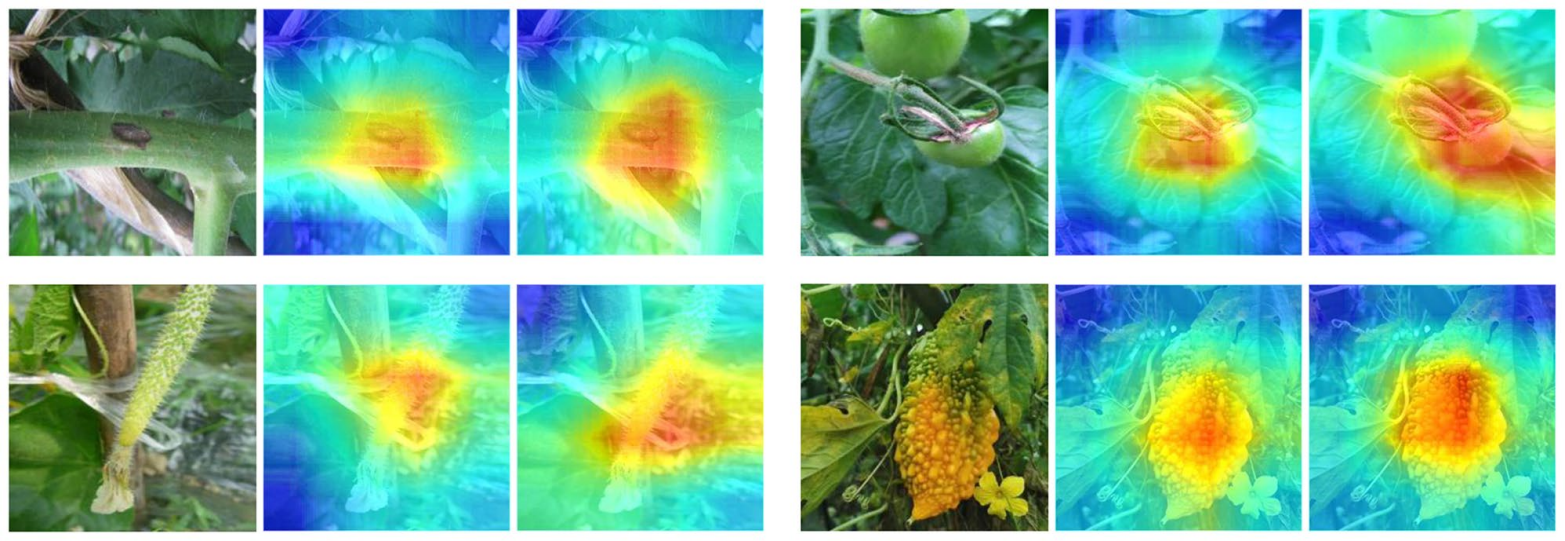
The feature attention heatmaps of four groups before and after adding the STFAN module

Figure 17 shows the feature attention heatmaps of four groups pre and post introduction of the STFAN module. The vegetable disease image in its original form is presented in the left column of every group. The attention heatmap prior to incorporating the module is displayed in the middle column, and the right column is the attention heatmap output after passing through the module. The darker the color, the greater the weight, which is more important for detecting vegetable diseases. Through this module, the network can focus on important areas and improve the performance of vegetable disease detection.

Although the core function of STFAN is classification, we unexpectedly observed a significant enhancement in the model’s focusing ability during the experiments. Specifically, STFAN uses a backbone network to extract multi-source information from the original vegetable disease images and outputs the type of vegetable to which the disease image belongs through a decision layer composed of two fully connected layers. This process not only improves the accuracy of classification but also enhances the model’s ability to focus on key features in the image. After the introduction of the STFAN module, the model’s attention heatmaps showed a clear preference for the disease feature areas, indicating that STFAN indirectly strengthens the model’s focus on important features when dealing with multi-source information. The experimental results show that STFAN not only effectively utilizes multi-source information for classification but also enhances the model’s ability to focus on key features, which is of great significance for improving the accuracy of vegetable disease detection. The improvement in focusing ability demonstrated by STFAN in classification tasks is due to its enhanced processing of image content during feature extraction and information fusion.

### Comparison of performance with and without multisource information

**Fig. 18:**
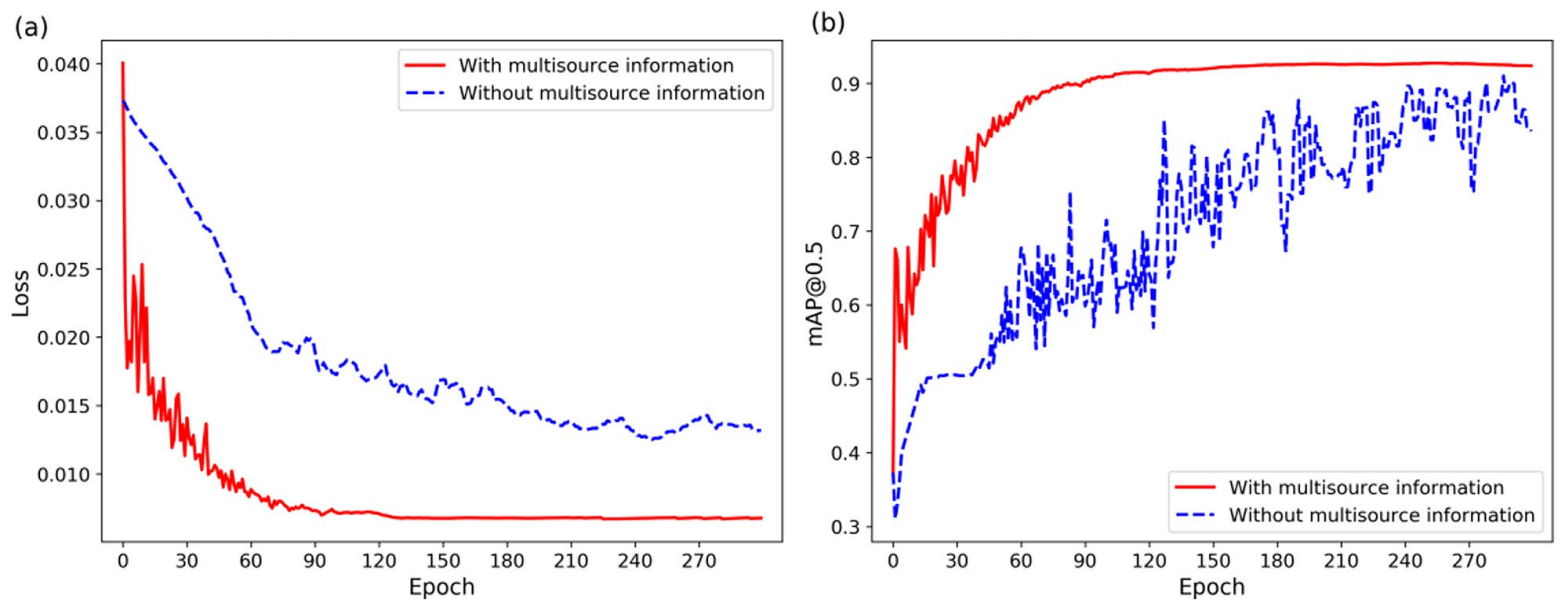
The mAP and loss curves during the training process with and without multisource information

To illustrate the effectiveness of multisource information, Table 8 presents the detection results of the MIFV algorithm with and without the use of multisource information, Fig. 18 presents the mAP and loss curves during the training process.

**Table 8:** Experimental comparison analysis with and without multisource information

Experiment	*P*(%)	*R*(%)	mAP(%)
Without multisource information	85.56	82.68	84.39
With multisource information	93.53	91.22	92.38

The data in Table 8 lead to the conclusion that employing multisource information enhances the performance of disease detection, nearly increasing by 9% compared to the use of disease images alone (from 84.39 to 92.38%). As shown in Fig. 18, the use of multisource information outperforms the non-use in terms of convergence speed and mAP.

Utilizing multisource information is more precise than relying on a single image modality. This underscores the complementary nature of visual information and textual descriptions in the identification process of diseased images. A single image modality extracts visual information from the image through a deep convolutional neural network and predicts its category. However, when the image contains few distinguishable features or is obscured by substantial noise, the addition of multisource information of the disease can enhance the complementarity between features, thereby facilitating the achievement of correct recognition outcomes.

## Conclusion and future research directions

### Conclusion

In response to the problem of insufficient utilization of multi-source information of vegetable diseases and large semantic gaps in feature fusion layers in current greenhouse vegetable disease target detection algorithms, which contain redundant information that interferes with the final detection effect, this study proposed the MIFV algorithm.The proposed MIFV algorithm redesigned and optimized the backbone network and feature fusion layers, improved the loss function. The structural design experiment of STFAN shows that multi-source information of vegetable diseases is crucial for detection. The design of MEDFFN indicates that suppressing deep redundant fusion features is also important. The BSLF loss function preserves the boundaries of vegetable disease targets well and effectively detects the contour of disease boundaries. Compared to the latest seven algorithms, the MIFV algorithm model has achieved significant improvement in detection performance on the VDGE dataset, which is conducive to improving the automation and intelligence level of greenhouse vegetable disease detection technology.

### Future research directions

This study proposes a Multisource Information Fusion Method for vegetable disease detection based on deep learning. By integrating multiple sources of information related to vegetable disease occurrence during visual extraction, it enhances the detection performance of vegetable diseases in complex scenarios, offering new research avenues in vegetable disease detection. However, there is still room for improvement.

1. As vegetables grow, the morphology of leaves and fruits undergo significant changes, leading to inconsistencies between test data and training set features. Future efforts should focus on long-term collection of vegetable disease image data at different growth stages. By dynamically updating training set data and exploring multi-layer interaction methods for multisource information of vegetable disease images, detection accuracy can be further improved.
2. In practical vegetable disease diagnosis, acquiring multisource information is challenging, and the effective information contained therein is unevenly distributed, leading to significant differences in the contribution of such information to the model. Therefore, future research should delve into the impact of missing or imbalanced multisource information on detection performance when inputting into the model.
3. Research on early detection of vegetable diseases remains to be explored, primarily due to the difficulty in collecting early disease image data. Early disease-infected areas exhibit fewer significant feature information, making it challenging for researchers to accurately detect disease types and infected areas. However, early disease detection is crucial for preventing the spread and development of disasters. Hence, future research should focus on early disease detection to achieve timely prevention and reduce losses.
4. We acknowledge that the MIFV algorithm may have limitations when dealing with rare diseases or low-quality images. Future work will explore improving the algorithm to enhance its performance in these scenarios.

## Data Availability

The data utilized in this paper is obtained through self-gathering and is made publicly available (a part of it) in order to make the study reproducible. It can be accessed by https://github.com/tyuiouio/plant-disease-detection-in-real-field. If you want to request the complete dataset and code, please email the corresponding author.

## Abbreviations

STFAN: Space-Time Fusion Attention Network
MEDFFN: Multilayer Encoder-Decoder Feature Fusion Network
BSLF: Boundary Structure Loss Function
MIFV: Multisource Information Fusion Method for Vegetable Disease Detection
VDGE: Vegetable Disease for Greenhouse Environment
